# Comparing the Movement System Impairment Method and Routine Physical Therapy for Knee Pain: A Randomized Clinical Trial

**DOI:** 10.3390/life15020179

**Published:** 2025-01-26

**Authors:** Mohammadreza Farazdaghi, Hassan Sadeghi, Marjan Alipour Haghighi, Salem M. Alshammari

**Affiliations:** 1 Department of Physical Therapy, School of Rehabilitation Sciences, Shiraz University of Medical Sciences, Shiraz 71348-14336, Iran; mfarazdagh@gmail.com; 2 Department of Biomechanics and Sports Injuries, Faculty of Physical Education and Sport Sciences, Kharazmi University, Tehran 15719-14911, Iran; 3 Department of Pharmaceutical Care, Alzahra and Hejazi Hospital, Shiraz University of Medical Sciences, Shiraz 71348-14336, Iran; m.alipour.h@gmail.com; 4 Department of Curriculum and Teaching Method, College of Education, Kuwait University, Kuwait City 31470, Kuwait; salemm.alshammari@ku.edu.kw

**Keywords:** exercise therapy, physiotherapy, knee pain, musculoskeletal pain, functional recovery

## Abstract

This study explores the effectiveness of the Movement System Impairment (MSI) model compared to traditional physiotherapy for treating knee pain. Fifty patients with unilateral knee pain participated, with their femur, tibia, and knee alignment assessed in nine functional positions. Evaluations included the Tegner Activity Scale, Knee Injury and Osteoarthritis Outcome Score (KOOS), muscle power, extensibility, and pain levels. Patients were randomly assigned to either the MSI treatment group, which focused on identifying and correcting faulty movements, or a routine physiotherapy group that received general strengthening and stretching exercises. Results indicated that both treatment approaches improved muscle power in hip abductors and lateral rotators, as well as scores on the Tegner Activity Scale and the KOOS. Notably, the MSI group demonstrated greater improvements in the muscle power of the hip lateral rotators and knee extensors and a significant reduction in knee pain during walking compared to the routine group (*p* = 0.005). In conclusion, both treatments enhanced pain, function, and muscle strength, while the MSI model significantly reduced knee pain in walking and improved hip and knee muscle power compared to routine physiotherapy.

## 1. Introduction

Knee pain is a prevalent musculoskeletal dysfunction that significantly impacts an individual’s functional ability throughout their life [1]. A considerable percentage, approximately 33%, of adults experience knee pain at some point, imposing a substantial burden on healthcare systems annually [2]. However, effectively treating knee pain remains a challenge due to conflicting outcomes derived from various outpatient care regimens [3]. The contentious nature of these treatments can be attributed to the inclusion of heterogeneous patient populations in research studies [3,4,5]. To address this issue, researchers have proposed different clinical classification methods, although only a few have undergone validation for knee pain impairments [3,6]. Among these methods, a limited number of models have investigated the role of faulty movement patterns in lower limb impairments and their subsequent modification [3,7].

Previous studies have recognized the modification of impairments as a fundamental aspect of treatment, albeit without utilizing a specific model. For instance, Salsich et al. employed task-specific training interventions to treat patellofemoral pain in patients, resulting in improved pain levels, functional capacity, and hip/knee kinematics during daily activities [8]. Keays et al. implemented an individualized treatment protocol based on impairments for patellofemoral pain patients, which yielded improvements in pain, muscle strength, and movement control [9]. Willy et al. specifically focused on modifying faulty movements, particularly during single-leg squatting and stair descending, which led to reductions in pain and functional improvements [10]. Noehren’s study on real-time gait training also demonstrated positive outcomes [11]. However, none of these studies presented a comprehensive and applicable model for diagnosing and treating knee pain.

The Movement System Impairment (MSI) model offers a diagnostic and therapeutic approach based on the classification of faulty movements and impairments. By considering signs and symptoms manifested during different activities, clinicians can diagnose specific impairments and formulate appropriate treatment plans [12]. The MSI model proposes seven categories of impairments, including tibiofemoral rotation (TFR) with valgus (TFRval) or varus (TFRvar), tibiofemoral hypomobility (TFHypo), knee hyperextension (HyperExt), knee extension, patellar lateral glide (PLG), tibiofemoral accessory hypermobility (TFAH), and knee impairments [7,13]. The model has demonstrated robust intertester reliability and satisfactory intratester reliability when applied to patients with knee pain. Although a new model has been proposed by Farazdaghi et al. based on the MSI model [14], the MSI model is still a widely accepted model among therapists.

The assessment process involves identifying the prevailing faulty movement pattern after evaluating patients in various positions [15], followed by repeating the assessment after modifying the faulty patterns in the same positions. If the symptoms are alleviated following the modification, it confirms the diagnosed syndrome. Recently, an RCT by Mousavi et al. compared the treatments of only TFR patients using the MSI model with routine physiotherapy and found patients had better alignments after treatment with the MSI model [16].

The necessity to evaluate the effectiveness of treating patients using the MSI model has been emphasized in the previous literature [8,9,13,16,17,18]. Therefore, the objective of this study is to investigate the effects of a 6-week treatment utilizing the MSI classification and treatment model on patients with knee pain to determine whether this model offers any superiority.

## 2. Materials and Methods

### 2.1. Participants and Study Design

This single-blinded randomized clinical trial study aimed to examine the efficacy of a therapeutic intervention for non-traumatic knee pain in patients. A convenience sampling method was employed to select the participants. Inclusion criteria stipulated that individuals should be aged between 18 and 65, have a minimum history of non-traumatic knee pain for two months before admission, and possess a standing Numerical Rating Scale (NRS) score ranging from 3 to 7. Eligible patients underwent initial screening conducted by an orthopedic physician, and those meeting the inclusion criteria were included in the study. Patients with orthopedic referrals for surgery, general acute metabolic/systemic diseases, neurologic diseases (e.g., radiculopathy), pregnancy, or recent use of analgesic and anti-inflammatory drugs were excluded. The sample size was determined as 40 cases based on previous studies by Salsich et al. and Keays et al. [8,9], aiming for a statistical power of 80% and an alpha error of 5%. All procedures used in this study followed the Declaration of Helsinki, and this research was approved by the ethics committee of The Sport Sciences Research Institute of Iran (IR.SSRC.REC.1401.067). It was also approved and registered in the Iranian Registry of Clinical Trials under the trial number IRCT20180627040251N5.

### 2.2. Procedure

Before participation, informed consent was obtained from the patients using a form developed by the Ethics Committee of Kharazmi University. The examination process comprised a comprehensive assessment involving history taking and physical examination. The history-taking phase included the collection of routine demographic parameters, pain history, and pain location. Furthermore, the Persian version of the Tegner questionnaire [19] was utilized to assess the patient’s current activity levels. It is a patient-administered activity-rating questionnaire for various knee disorders with acceptable test/retest reliability, floor and ceiling effects, criterion validity, construct validity, and responsiveness [20]. To measure pain, symptoms, and knee function, the Knee Injury and Osteoarthritis Outcome Score (KOOS) questionnaire was administered [21]. It is a valid and reliable questionnaire (ICC = 0.70) with acceptable responsiveness [22,23] consisting of 5 subscales. In cases where patients presented with knee ligament laxity or injury, such information was recorded during the history assessment. In the initial stage, the patients’ faulty alignments (signs) were visually evaluated in various positions, such as standing, sitting, walking, single-leg standing, standing from a sitting position, partial squatting, and climbing stairs. The visual assessment of lower extremity impairments is a valid and reliable assessment method (substantial intra-rater and fair to substantial inter-rater agreement) for the evaluation of lower extremity biomechanical problems [9,24]. Pain intensity (symptoms) was quantified using the Numerical Rating Scale (NRS). Manual muscle testing (MMT) tests were conducted to evaluate muscle strength of hip, knee and ankle muscles by Kendall’s method [25,26]. This method scores the muscle power from 0 to 5 and has been shown to have a good internal and external validity and reliability [26]. Furthermore, the muscle extensibility of the quadriceps, hamstrings, gastrocnemius, and tensor fascia lata/iliotibial band (TFL-ITB) was assessed using goniometry following the method described by Kendall [25] to evaluate relative flexibility. Quadriceps extensibility was measured by assessing the range of motion (RoM) at the knee while the patient lay prone. The examiner passively bent the patient’s knee, recording the angle of flexion. Hamstring extensibility was assessed with the patient lying supine. The examiner bent the patient’s hip and knee to 90 degrees and then passively extended the knee, recording the RoM of knee extension. TFL-ITB extensibility was assessed with the patient in a side-lying position, using goniometry to measure the degree of stretch during passive hip adduction. Gastrocnemius flexibility was evaluated by passively dorsiflexing the ankle with the patient in a supine position and measuring the ankle’s RoM.

**Figure 1:**
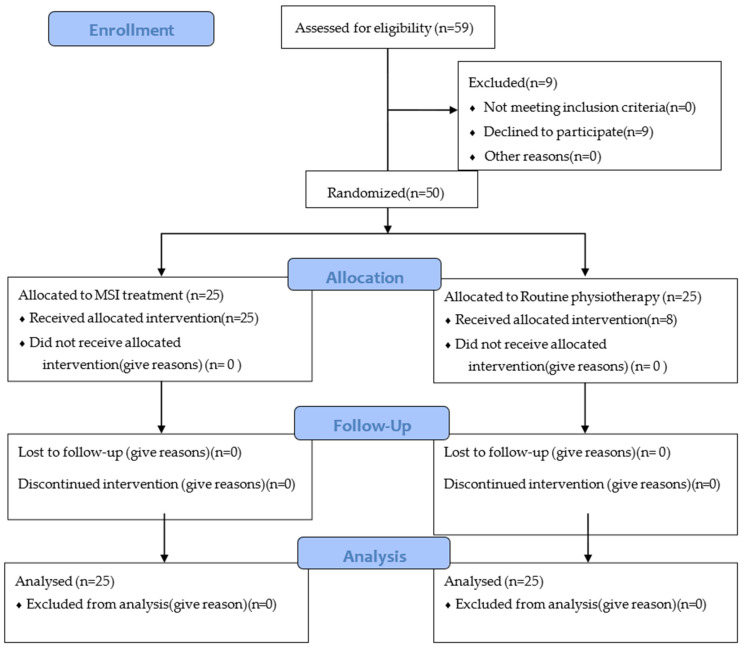
CONSORT flowchart of participant recruitment.

Then, patients were randomly assigned to one of the groups (MSI group or routine physiotherapy group) using the Research Randomizer program (Research Randomizer, version 4, Geoffrey C. Urbaniak, Lancaster, PA, USA). Allocation was concealed as the ResearchRandomizer program performed the randomization. Patients were blinded to their group assignment, and only the examiner responsible for conducting the assessment and treatment was aware of the group allocation. The CONSORT flowchart summarizes information about the study population, samples, and analysis (Figure 1). All procedures were conducted according to the Declaration of Helsinki.

### 2.3. MSI Group Protocol

Following the initial assessments, the examiner identified and explained the patients’ faulty positions during various movements and provided corrections to improve their movement patterns accordingly. The examiner then repeated the assessment and documented any changes in symptoms. The MSI model was employed to assess the alignment of the tibia, femur, and knee in sagittal, coronal, and frontal planes for different movements (Supplementary S1). Positive findings were recorded to identify the presence of syndromes. Based on the observed signs and symptom changes, the therapist performed a judgement and assigned a specific syndrome to each patient using the MSI model (Supplementary S1) [27]. Individualized treatment was initiated, tailored to the assigned syndrome, and adjusted according to the patient’s condition over a 6-week period, with one session per week. During these sessions, the modification of daily activities, such as sit-to-stand and stair climbing and descending, was explained to the patients. Strengthening and conditioning exercises were prescribed to improve the patients’ condition. In cases where excessive pronation or supination of the foot was observed, a semi-rigid prefabricated foot orthosis was recommended. To assist the patients in their self-care between sessions, they were provided with detailed notes outlining their specific impairments and reminding them of the exercises they needed to perform. The exercises and modifications of movements were adjusted based on the patients’ abilities and pain levels, with the primary goal of improving their condition without exacerbating pain [27]. Supplementary S2 shows the MSI treatment protocol that was used for the MSI group.

### 2.4. Routine Physiotherapy Group

Strengthening exercises and stretching of hip and knee muscles were given to patients. In cases where excessive pronation or supination of the foot was observed, a semi-rigid prefabricated foot orthosis was recommended. Individualized treatment was initiated and adjusted according to the patient’s condition over 6 weeks [28], with one session per week. Some general tips about knee RoM during movements were given as well. To assist the patients in their self-care between sessions, they were provided with notes outlining the exercises they needed to perform. The exercises and modification of movements were adjusted based on the patients’ abilities and pain levels, with the primary goal of improving muscle power of knee and hip muscles without exacerbating pain [27]. The exercises were advanced to include resistance bands or weights if the patient reported that they were too easy. Initially, the exercises were performed once daily and were gradually increased to three times per day based on the patient’s ability to progress.

After six weeks, the patients’ pain, symptoms, function, activity level, muscle strength, and muscle extensibility were reassessed regardless of their group.

### 2.5. Statistical Analysis

The data obtained from the study participants were subjected to statistical analysis using SPSS version 25 software (IBM Inc., Chicago, IL, USA). To assess the normal distribution of the measured variables, the Kolmogorov–Smirnov test was employed. Within-group analyses were performed using the Wilcoxon test for non-parametric variables, while between-group analyses were conducted using the Mann–Whitney test. The level of statistical significance was set at *p* < 0.05, indicating that differences with a probability of less than 5% were considered statistically significant.

## 3. Results

Fifty patients entered the study and were evaluated again after a 6-week period. Demographic characteristics of these patients are shown in Table 1. Table 2 demonstrates these characteristics in patients categorized with the MSI model (MSI group).

Within-group and between-group analyses were performed on the findings.

### 3.1. Within-Group Analysis

The Wilcoxon signed-rank tests were conducted to assess changes in various outcome measures pre- and post-treatment within each patient group. Among the 29 variables analyzed, significant differences were observed in both groups.

The patients who received MSI treatment showed significant changes in the muscle power of the hip abductors (*p* = 0.005), hip lateral rotators (*p* = 0.02), and ankle plantar flexors (*p* = 0.04), along with KOOS scores for different components of pain (0.002), symptoms (*p* = 0.004), ADL (*p* = 0.000), sport (*p* = 0.001), and quality of life (*p* = 0.001). Also, the Tegner score (0.01), ankle dorsiflexion RoM (*p* = 0.006), and hip abductor MMTs (*p* = 0.01) were different.

Patients who were treated with routine exercises had significant differences in the muscle power of the hip abductors (*p* = 0.01), hip lateral rotators (*p* = 0.01), hip extensors (*p* = 0.01), along with KOOS scores for different components of pain (*p* = 0.001), symptoms (*p* = 0.033), ADL (*p* = 0.000), and sport (*p* = 0.009). The Tegner score (*p* = 0.002), knee pain during walk (*p* = 0.000), and pain during sit-to-stand (*p* = 0.01) were also different, as well as hip abductor extensibility (*p* = 0.005).

### 3.2. Between-Group Analysis

Further analyses involved comparing the distributions of the aforementioned significant variables among the groups using the Mann–Whitney test. The results unveiled significant differences in the muscle power of the hip lateral rotators (*p* = 0.02) and knee extensors (*p* = 0.01) and knee pain during walk (*p* = 0.03) (Table 3).

## 4. Discussion

This study’s results demonstrate comparable improvements across various parameters for both groups, irrespective of whether they engaged in MSI-specific exercises or routine physiotherapy [1]. The reason that we could not find expected major differences between the two protocols is likely due to the similarity of the treatment methods, as also shown by Mousavi et al. in 2024 [16]. In fact, both models include similar stretching and strengthening exercises, which could explain why both groups demonstrated comparable results (Supplementary S2). However, a significant divergence emerges in the muscle powers of the knee extensors and hip lateral rotators, essential muscles in knee pain treatment, as well as knee pain during walking.

Prior research consistently supports the positive effects of muscle strengthening on alleviating pain and improving function in individuals with knee pain [29,30,31]. Notably, the focus on strengthening hip lateral rotators and abductors stands out as a recommended approach for treating patellofemoral pain due to their stabilizing effects on the patellofemoral joint and enhancement of knee function during various activities [32,33]. In fact, there is a positive correlation between patellofemoral pain with hip muscle weakness [32]. Quadriceps strengthening is an accepted standard treatment for improving function in patients with PFP. Recent studies further suggest that hip muscles are more effective than knee-extensor strengthening in treating patellofemoral pain, but a combination of hip/quad exercises has been introduced as a preferable approach over strengthening each compartment in isolation [32].

Significant improvements in muscle power within the MSI group, compared to routine physiotherapy, may be attributed to the MSI system’s focus on correcting extremity and joint alignment and identifying muscle weaknesses [16]. Weak hip abductors and lateral rotators can result in femoral internal rotation, contributing to increased involvement and pressure on the lateral patellar and femoral tissues [34]. This aligns with studies showing that hip abductor strength-based exercises can enhance performance and self-reported function, particularly in women with symptomatic knee osteoarthritis (OA) [35]. A review by Neelapala et al. identified strong, high-quality evidence to recommend hip-muscle strengthening in the conservative management of people with knee OA [36]. In fact, studies have mentioned improvement of pain and function with correction of the hip and knee kinematics [11,37]. Aligning with the MSI model’s approach, a new systematic review and meta-analysis by Yokoyama emphasized the combination of exercise therapy with biomechanical intervention to decrease the peak of the adduction moment in movements [38].

In the current study, although exercises were matched with the patient’s current abilities, the focus of exercises was mainly on self-correction of impairments and strengthening of some specific muscles. Modifications were adjusted based on the patients’ syndromes through the MSI model. For example, regarding TFR syndrome, the modifications included activation of the gluteal muscles to prevent femoral adduction/medial rotation in movements and modification of daily activities [27,39] since such rotation was recorded in over 72% of patients. The recognition that impairments such as femoral medial rotation are commonly reported in patients with knee pain adds further support to the significance of these modifications [27].

The findings of the current study are in line with those of previous studies [8,9,40,41,42,43,44] except for Salsich et al. in 2010. Salsich’s study showed that although correction of valgus impairment in patellofemoral pain patients could decrease the femoral medial rotation and knee lateral rotation, it could not significantly decrease the patients’ symptoms after [40]. In fact, they evaluated the immediate effect of alignment correction and recorded the pain in that session. They also mentioned the pain decrement that we expect might happen with the improvement of performance and tissue healing over time with exercises [28,45]. Smith et al. performed a 12-week exercise and education program on hip and knee osteoarthritic patients and found that it could improve function and pain and decrease stiffness and increase self-reported wellbeing [41]. Another study by Bhore et al. found the same result as multi-directional exercise can improve gait parameters, pain, and strength in patients with knee OA [42]. A new systematic review by Holden et al. proposed that patients that have OA and attend with higher pain and physical function scores may gain more benefits from exercise in the short term [43]. In 2018, Salsich et al. found that 6 weeks of training patients with patellofemoral pain can decrease the pain and increase their functioning. The pain reduction was significant after 12 weeks of training [8]. In the current study, it took only six weeks to obtain the same satisfactory results, except that the patients had different syndromes based on the MSI model other than patellofemoral pain. Our findings indicate that even in the shorter duration, MSI-specific exercises focusing on correcting movement impairments and strengthening targeted muscle groups can achieve comparable outcomes in pain reduction and functional improvement. The relatively rapid improvements in our study may be attributed to the individualized nature of the MSI approach, which tailors interventions to specific biomechanical impairments. However, it is important to recognize that some benefits, such as sustained tissue adaptation and long-term biomechanical alignment, might manifest more prominently with extended intervention periods. For example, Smith et al.’s 12-week program improved self-reported wellbeing and physical function significantly more than shorter programs [41], underscoring the importance of long-term adherence to therapeutic exercises. Similarly, Salsich et al. highlighted that the immediate correction of impairments does not always result in significant symptom reduction unless supported by sufficient time for tissue adaptation and healing [40]. In the context of our study, the 6-week duration was sufficient to achieve clinically meaningful improvements, as evidenced by KOOS subscale scores meeting or exceeding the minimal detectable change (MDC) thresholds. This aligns with findings from shorter-duration interventions in other studies, such as Keays et al. [9], which showed significant progress within 4 weeks. Nevertheless, our results suggest that extending the intervention period may further enhance long-term outcomes, particularly in patients with chronic conditions or more complex impairments.

In a study by Keays et al. in 2015, the strength and tightness of the quadriceps, pain, and knee control improved after four weeks of individual treatment in patients with patellofemoral pain [9]. Throughout the study, they clustered the patients with similar dysfunctions into four main groups of hypermobility, hypomobility, faulty movement patterns, and patellofemoral osteoarthritis. These might be the reason for the significant pain difference that was found between both groups in the current study. Zamani et al. performed a study and classified knee pain patients into two groups, the MSI group and control group, but performed routine physiotherapy on both groups [44]. The results showed improvement of symptoms and KOOS and lower extremity functional scale scores in both groups and gave better insight to the therapist, and they mentioned that specific treatments based on the movement impairment classification have beneficial effects over routine treatment, which might be a vague statement since both groups received the same treatment. In the current study, we gave each MSI group a specific exercise regarding those patients and compared the outcomes with the routine physiotherapy treatment group.

The minimal important difference for the pain subscale of the KOOS questionnaire for knee injuries is between 6 and 13.4 points [21]. Also, the MDC for different items of the KOOS questionnaire has been reported as follows: pain: 6–6.1, symptoms: 5–8.5, ADL: 7–8, Sport/rec: 5.8–12, and QoL: 7–7.2 [21]. As to the MDC value, all improvements measured by the KOOS showed a clinically significant reduction after 6 weeks in both groups. Since the MDC for the Tegner score has been reported as 1 [46], the improvement in the patients both groups is not clinically significant after 6 weeks.

Finally, this study had a number of important limitations which should be considered. Firstly, we did not measure the amount of the patients’ adherence to exercises. Low levels of adherence may have affected the amount of muscle power or flexibility improvement. The feasibility of a movement training program on patients with knee pain has been approved previously [8]. Similar studies have reported adherence of greater than 75% [8] and 55–69% [46]. Secondly, we only compared the short-term effects, and long-term effects should be evaluated in future studies. As the study of Hott et al. showed, there is no difference between the long-term exercise of the hip or knee or free activity, which might question the long-term effects of these exercises [47]. Another study evaluates and compares the effectiveness of medial versus lateral injection techniques for administering hyaluronic acid in patients with knee osteoarthritis, highlighting the implications for optimizing pain management strategies [48]. We suggest that future studies focus on evaluating the long-term effects of the MSI approach on patients with knee pain. Additionally, as this study did not assess patient adherence to the prescribed treatment, future research should incorporate methods to measure adherence to better understand its impact on outcomes.

## 5. Conclusions

This study is one of the first to directly compare the movement system impairment (MSI) approach with routine physiotherapy protocols in the management of unilateral knee pain, highlighting the unique advantages of the MSI model. Specifically, our findings demonstrate that the MSI approach not only reduces pain during walking effectively but also significantly improves muscle power in both the hip and knee after six weeks of intervention. This study provides clinical evidence supporting the integration of the MSI treatment model into rehabilitation protocols for knee pain, offering enhanced functional outcomes and greater effectiveness in addressing underlying biomechanical impairments. However, both the MSI and routine physiotherapy approaches significantly improved key functional and pain parameters, with no significant difference observed between them, which may call into question the superiority of the MSI model. Future research with larger cohorts and extended follow-up periods is needed to validate these findings and assess their broader applicability.

## Figures and Tables

**Table 1:** Demographic characteristics of all patients and different groups of the study.

	All	Movement System Impairment Model	Routine Physiotherapy
Age	44.12 ± 12.41	43.12 ± 13.83	45.15 ± 10.99
Sex	Male: 13 Female: 37	Male: 7 Female: 18	Male: 6 Female: 19
Weight (Kg)	73.60 ± 13.75	73.16 ± 13.90	74.04 ± 13.86
Height (Cm)	167.04 ± 9.04	168.28 ± 9.38	165.80 ± 8.70
Number of participants	50	25	25
Affected knee	Left: 25 Right: 25	Left: 11 Right: 14	Left: 14 Right: 11
Current pain (Numeric Rating Scale)	17.10 ± 20.15	14.00 ± 21.93	20.20 ± 18.11

**Table 2:** Demographic characteristics of patients in MSI group based on their sub-categories.

	Categorized Based on Movement System Impairment Model				
	TFRval	TFRvar	TFHypo	PLG	TFAH
Age	41.78 ± 15.46	51.75 ± 10.21	59.67 ± 0.57	36.29 ± 9.84	31.00 ± 5.65
Sex	Male: 1 Female: 8	Male: 2 Female: 2	Male: 1 Female: 2	Male: 2 Female: 5	Male: 1 Female: 1
Weight (Kg)	71.56 ± 12.20	77.75 ± 12.76	76.67 ± 16.50	71.00 ± 19.20	73.50 ± 4.95
Height (Cm)	166.67 ± 6.08	166.00 ± 8.08	169.00 ± 11.53	167.71 ± 12.80	181.00 ± 1.41
Number of participants	9	4	3	7	2
Affected knee	Left: 5 Right: 4	Left: 2 Right: 2	Left: 1 Right: 2	Left: 3 Right: 4	Left: 0 Right: 2
Current pain (NRS)	16.67 ± 29.89	25.00 ± 23.80	10.00 ± 17.32	10.00 ± 12.91	0.00 ± 0.00

TFRval: tibiofemoral rotation with valgus, TFRvar: tibiofemoral rotation with varus, TFHypo: tibiofemoral hypomobility, PLG: patellar lateral glide, TFAH: tibiofemoral accessory hypermobility.

**Table 3:** Between-group analysis of measured parameters before intervention and after intervention.

	Measured Variable	Status	Mean ± SD		*p*-Value
			MSI Group	Routine Group	
Relative flexibility	Ankle Dorsiflexion RoM	Primary visit	17.56 ± 7.37	18.48 ± 6.60	0.77
		Last Visit	18.92 ± 6.22	19.20 ± 6.00	0.83
	Knee Extension RoM	Primary Visit	79.80 ± 5.18	82.08 ± 6.18	0.09
		Last Visit	80.08 ± 5.09	81.80 ± 5.62	0.17
	Hip Adduction RoM	Primary Visit	9.32 ± 2.34	9.08 ± 1.65	1.0
		Last Visit	9.96 ± 1.85	9.92 ± 1.46	0.72
	Knee Flexion RoM	Primary visit	11.96 ± 2.63	12.04 ± 3.46	0.74
		Last Visit	11.94 ± 2.53	11.82 ± 2.93	0.56
Manual Muscle Testing (MMT)	MMT HIP ABD	Primary visit	4.08 ± 0.70	4.28 ± 0.61	0.31
		Last Visit	4.40 ± 0.50	4.52 ± 0.51	0.39
	MMT HIP ADD	Primary Visit	4.00 ± 0.86	3.92 ± 0.75	0.74
		Last Visit	4.04 ± 0.84	3.96 ± 0.73	0.71
	MMT Hip Lat Rot	Primary visit	4.20 ± 0.50	4.24 ± 0.66	0.70
		Last Visit	4.16 ± 0.47	4.48 ± 0.51	0.02 *
	MMT Hip Med Rot	Primary Visit	4.04 ± 0.67	4.24 ± 0.59	0.28
		Last Visit	4.04 ± 0.61	4.24 ± 0.59	0.16
	MMT Hip Flex	Primary Visit	4.36 ± 0.49	4.36 ± 0.56	0.91
		Last Visit	4.40 ± 0.50	4.40 ± 0.50	1.00
	MMT Hip Ext	Primary Visit	4.08 ± 0.75	3.84 ± 0.55	0.21
		Last Visit	4.28 ± 0.67	4.24 ± 0.43	0.62
	MMT knee Ext	Primary Visit	4.20 ± 0.40	4.24 ± 0.52	0.70
		Last Visit	4.08 ± 0.40	4.44 ± 0.58	0.01 *
	MMT Knee Flex	Primary Visit	3.96 ± 0.53	4.00 ± 0.50	0.78
		Last Visit	4.00 ± 0.57	4.00 ± 0.50	1.00
	MMT Ankle PF	Primary Visit	4.76 ± 0.52	4.84 ± 0.37	0.67
		Last Visit	4.92 ± 0.27	4.88 ± 0.33	0.64
KOOS Questionnaire	KOOS Pain	Primary Visit	66.88 ± 18.08	69.80 ± 17.84	0.52
		Last Visit	77.56 ± 21.58	84.40 ± 10.21	0.43
	KOOS Sym	Primary Visit	51.48 ± 14.93	57.04 ± 13.65	0.11
		Last Visit	59.12 ± 10.05	63.20 ± 6.21	0.18
	KOOS ADL	Primary Visit	77.52 ± 14.47	79.44 ± 13.82	0.43
		Last Visit	88.32 ± 11.76	92.28 ± 6.44	0.31
	KOOS Sport	Primary Visit	37.60 ± 24.28	49.80 ± 27.85	0.10
		Last Visit	56.04 ± 24.66	62.80 ± 20.16	0.33
	KOOS QoL	Primary Visit	44.92 ± 11.48	49.04 ± 19.25	0.20
		Last Visit	51.60 ± 16.81	50.68 ± 15.28	0.89
Tegner	Tegner Score	Primary Visit	4.92 ± 1.47	4.88 ± 1.85	0.76
		Last Visit	5.52 ± 1.32	5.56 ± 1.63	0.87
NRS	Pain while Walking	Primary Visit	19.40 ± 19.80	21.20 ± 15.56	0.61
		Last Visit	10.80 ± 18.12	21.00 ± 20.56	0.03 *
	Pain with Sit-to-Stand	Primary Visit	11.40 ± 21.28	7.00 ± 15.67	0.20
		Last Visit	7.00 ± 12.33	4.00 ± 10.80	0.93

RoM: Range of Motion, ABD: Abduction, ADD: Adduction, Lat Rot: Lateral Rotation, Med Rot: Medial Rotation, Flex: Flexion, Ext: Extension, PF: Plantar Flexion, Sym: Symptom, ADL: Activities of Daily Living, QoL: Quality of Life, KOOS: Knee Injury and Osteoarthritis Outcome Score * *p* value < 0.05.

## Data Availability

The datasets used and/or analyzed in the current study are available from the corresponding author upon reasonable request.

## Supplementary Materials

The following supporting information can be downloaded at: https://www.mdpi.com/article/10.3390/life15020179/s1, Supplementary S1: Evaluation list of different MSI syndromes; Supplementary S2: List of exercises for different MSI syndromes.
