# Supplementary material for: Comparing the Movement System Impairment Method and Routine Physical Therapy for Knee Pain: A Randomized Clinical Trial
Source: Life (Basel). 2025 Jan 26;15(2):179. doi: 10.3390/life15020179 (PMC11856088; doi:10.3390/life15020179)
Supplement: Supplementary file 1 [file life-15-00179-s001.zip › Supplementary S1.pdf]

Supplementary S1:

Evaluation list of different MSI syndromes

1A: Standing (Sign)	
Front/Back view:	
vertical crease prominence	Inner Outer
Femoral medial rotation/adduction	Yes No
Tibial external rotation	Yes No
Knee	Valgus Varus No Impairment
Foot	Supination Pronation No Impairment
Lateral view:	
Lack of full extension	Yes No
Knee hyperextension	Yes No
Posture (Mark if Sway back, military, kyphotic-lordotic, flat back)	
1B: Standing (symptom)	
Numeric Rating Scale (0-10)	
2A: Single leg standing on affected leg (Sign)	
Symptoms compared to standing	Increased Decreased No change
Numeric Rating Scale (0-10)	
Did modification decrease the symptoms?Numeric Rating Scale (0-10)	Yes No ……………………
2B: Single leg standing on affected leg (symptom)	
Tibial external rotation (Foot twisting out)	Yes No
vertical crease twisted	Clockwise Counterclockwise
3A: Bending the affected knee 90 while standing single leg on affected leg (Sign)	
Tibial external rotation (Foot twisting out)	Yes No
vertical crease twisted	Clockwise Counterclockwise
3B: Bending the affected knee 90 while standing single leg on affected leg (Symptom)	
Symptoms compared to standing	Increased Decreased No change
Numeric Rating Scale (0-10)	
Did modification decrease the symptoms?Numeric Rating Scale (0-10)	Yes No ……………………
4A: Partial squat (Sign)	
Femoral medial rotation/adduction	Yes No
Tibial external rotation	Yes No

Knee	Valgus Varus No Impairment
4B: Partial squat (Symptom)	
Symptoms compared to standing	Increased Decreased No change
Numeric Rating Scale (0-10)	
Did modification decrease the symptoms?Numeric Rating Scale (0-10)	Yes No …………………….
5A: Prone active knee flexion (Sign)	
Tibial external rotation	Yes No
Knee	Valgus Varus No Impairment
5B: Prone active knee flexion (Symptom)	
Symptoms compared to standing	Increased Decreased No change
Numeric Rating Scale (0-10)	
Did modification decrease the symptoms?Numeric Rating Scale (0-10)	Yes No …………………….
6A:Sit-to-stand (Sign)	
Femoral medial rotation/adduction	Yes No
Tibial external rotation	Yes No
Knee	Valgus Varus No Impairment
6B: Sit-to-stand (Symptom)	
Symptoms compared to standing	Increased Decreased No change
Numeric Rating Scale (0-10)	
Did modification decrease the symptoms?Numeric Rating Scale (0-10)	Yes No …………………….
7A:Step up (Sign)	
Femoral medial rotation/adduction	Yes No
Tibial external rotation	Yes No
Knee	Valgus Varus No Impairment
7B: Step up (Symptom)	
Symptoms compared to standing	Increased Decreased No change
Numeric Rating Scale (0-10)	
Did modification decrease the symptoms?Numeric Rating Scale (0-10)	Yes No …………………….
8A:Step down(Sign)	
Femoral medial rotation/adduction	Yes No
Tibial external rotation	Yes No
Knee	Valgus Varus No Impairment
8B:Step down (Symptoms)	
Symptoms compared to standing	Yes No
Numeric Rating Scale (0-10)	Yes No

Did modification decrease the symptoms?Numeric Rating Scale (0-10)	Valgus Varus No Impairment
9A:Sitting (Sign)	
Femoral medial rotation/adduction	Yes No
Tibial external rotation	Yes No
Knee	Valgus Varus No Impairment
9B:Sitting (Symptoms)	
Symptoms compared to standing	Increased Decreased No change
Numeric Rating Scale (0-10)	
Did modification decrease the symptoms?Numeric Rating Scale (0-10)	Yes No …………………….
10: Testing muscle extensibility	
Quadriceps	Yes No …………………….
TFL-ITB	Yes No …………………….
Hamstring	Yes No …………………….
Gastrocsoleus	Yes No …………………….
11: Manual Muscle Testing	
Hip abductor group	…………………….
Hip extensor group	…………………….
Hip flexor group	…………………….
Hip lateral rotator group	…………………….
Hip adductor group	…………………….
Hip medial rotator group	…………………….
Knee extensor group	…………………….
Knee flexor group	…………………….
Ankle plantar flexor group	…………………….
