# Supplementary material for: Comparing the Movement System Impairment Method and Routine Physical Therapy for Knee Pain: A Randomized Clinical Trial
Source: Life (Basel). 2025 Jan 26;15(2):179. doi: 10.3390/life15020179 (PMC11856088; doi:10.3390/life15020179)
Supplement: Supplementary file 1 [file life-15-00179-s001.zip › Supplementary S2.pdf]

Supplementary S2:

List of exercises for different MSI syndromes

Perform only the ones that the therapist has marked to avoid confusion.

1-Tibiofemoral rotation syndrome

A. To improve alignment deficit:

a. Unlock knees if you do.
b. Align knees over feet in different movements.
c. Pay attention to your thigh and leg. Prevent excessive rotations in different movements such as part “b”.
d. Do not perform part “c” if the therapist talks to you about a structural deficit. Instead, it is okay to have rotations in thigh and foot.

B. Gait

a. Unlock knees if you do and raise your heel when taking off your foot.
b. Do not let your thigh rotate medially when you are bearing weight on your feet. To achieve this contract the buttock muscles on exercises as follow:
    i. Stand upright with feet apart. Shift weight on one foot and contract the buttock muscles.
    ii. Stand upright in semi-tandem position. Shift weight on one foot and contract the buttock muscles.
    iii. Stand upright in semi-tandem position. Shift weight on one foot and contract the buttock muscles.
    iv. Walk and contract the buttock muscles as you shift weight.
c. Do not walk with the sole of foot. Your foot contacts the ground from heel and takes off from toes.

d. If the therapist talks to you about having sever bow leg, you can walk with rotation of your thigh outward.
e. You are free to use a cane if necessary. Ask your therapist the preferred side of using cane.

C. Sit-to-standing

a. Slide forward in chair. Align feet behind knees as hip-with distance. Contract your buttock and front thigh muscles to lift body up and forward out of chair.
b. Do not forget to let your tibia roll over your foot.
c. Avoid hip medial rotation.
d. Avoid pulling knees back to meet body.

D. Ascending stairs

a. Use rail to decrease weight on the affected limb.
b. Contract your buttock and front thigh muscles to lift body up and forward
c. Do not let your thigh rotate medially when you are bearing weight on your feet.
d. Avoid pulling knees back to meet body.

E. Descending stairs

a. Use rail to decrease weight on the affected limb.
b. Do not let your thigh rotate medially when you are bearing weight on your feet.
c. If it is difficult, step down with the involved extremity.
d. If it is difficult too, descend backwards.

F. Daily activities

a. The therapist will talk to you about daily routines.

G. Running

a. First learn to walk with instructions. Then gradually start running in short periods and increase the time of running.

b. Start running on track or chip trail instead of asphalt. Avoid running on concrete on camber.

c. Shift your weight slightly forward while running.

H. Cycling

a. It’s better to use toe clips.

I. Home exercise

Exercise for lateral buttock and intrinsic muscles

You should not feel an increase in symptoms or experience a pressure in the knee during exercise. If so, contact your therapist. In all of the exercises you should feel the contraction in your buttock.

a. Sleep on your belly. Bend your knees to 90 degrees. Push your feet together.

b. Sleep on your belly with knees straight. Now move one entire leg away from the other one.

c. Sleep on your side with knees slightly bent. Now move the upper leg away wile the feet are together.

d. Sleep on your side with knees straight. Spin the thigh around an axis longitudinally through the femur.

e. Perform the previous exercise while moving the upper leg up from the ground.

f. Perform previous exercise with elastic band.

g. Perform exercise “d” with elastic band.

h. Sit on a chair. Push your feet together. Feel the contraction in your buttock.

i. Stand. Push your feet together. Feel the contraction in your buttock.

j. Perform lunges with good alignment of lower extremities.

k. Perform lunges with elastic band.

l. Perform previous exercise while holding a weight.

Exercise for buttock muscles

a. Sleep on your belly with a pillow under pelvis. Bend your knees to 90 degrees. Control the movement of your leg and push the entire leg toward the ceiling.
b. Perform squatting.
m. Perform squatting while holding a weight.

Exercise abdominal muscles

n. Pull in your abdominal muscles with functional activities.
o. The therapist may give you some abdominal exercises if needed.

J. Exercise to improve extensibility

Stretching of lateral and anterior parts of thigh

a. Make sure your pelvis is in good position and not tilted forward.
b. Sleep on your belly with a pillow under pelvis. Bend your knees at the same time and avoid leg rotation. If it is painful start the movement with thighs apart.
c. Sleep near the end of the bed. Flex both knees to your chest. Now release on leg and extend it as far as it goes. Let your thigh move outward but control your leg not to move. You should gradually be able to close and control your thigh actively. Avoid rotation of the leg or slightly point it inward.
d. Sleep on your belly with a pillow under pelvis. Bend one knee and rotate your thigh so that your leg moves toward the other leg. Avoid leg rotation.
e. Sleep near the edge of your bed at your side. Now with your knee straight move the entire leg back and drop it from behind (drop from the bed). You should feel the stretch in lateral side of buttock and thigh.

Stretching of back of thigh and foot

a. Sit on a chair. Actively extend your knees while pulling feet toward yourself.
b. Sleep on your back. Flex your hip to 90 degrees. Now extend your knee.

c. Stand in runners’ position while your heel is on the ground. Feel the stretch in your leg.

K. Taping

a. Posterior X-taping will be performed based on the therapist’s opinion and aims to control rotation of the thigh. Other forms of taping may be performed as well. In case of itching, irritation and skin blisters remove the tape or call your therapist.

L. Bracing and orthotics

a. If the therapist advices a brace or shoe insert, please use them regularly.

M. Pain control

a. You can use ice to control pain as needed.

b. Modify activities to reduce stress.

N. Neuromuscular training

a. Ask your therapist about sport-specific trainings, drills, running, jumping, and agility exercises. You will receive another note for such exercises.

Perform only the ones that the therapist has marked to avoid confusion.

2-Tibiofemoral hypomobility syndrome

a. **To improve alignment deficit:**
    a. Pay attention to what the therapist tells you about your posture.
b. **Gait**
    a. Do not let your thigh rotate medially when you are bearing weight on your feet. To achieve this contract the buttock muscles on exercises as follow:
        i. Stand upright with feet apart. Hold your hand on a place to decrease weight on feet. Shift weight on one foot and contract the buttock muscles.
        ii. Stand upright in semi-tandem position. Hold your hand on a place to decrease weight on feet. Shift weight on one foot and contract the buttock muscles.
        iii. Stand upright in semi-tandem position. Hold your hand on a place to decrease weight on feet. Shift weight on one foot and contract the buttock muscles.
        iv. Walk and contract the buttock muscles as you shift weight.
    b. Do not walk with the sole of foot. Your foot contacts the ground from heel and takes off from toes.
    c. If the therapist talks to you about having sever bow leg, you can walk with rotation of your thigh outward.
    d. You are free to use a cane if necessary. Ask your therapist the preferred side of using cane.

O. **Sit-to-standing**
    a. Slide forward in chair. Align feet behind knees as hip-with distance. Contract your buttock and front thigh muscles to lift body up and forward out of chair.
    b. Do not forget to let your tibia roll over your foot.
    c. Avoid hip medial rotation.

d. Avoid pulling knees back to meet body.

P. Ascending stairs

a. Use rail to decrease weight on the affected limb.

b. Contract your buttock and front thigh muscles to lift body up and forward

c. Do not let your thigh rotate medially when you are bearing weight on your feet.

d. Avoid pulling knees back to meet body.

Q. Descending stairs

a. Use rail to decrease weight on the affected limb.

b. Do not let your thigh rotate medially when you are bearing weight on your feet.

c. If it is difficult, step down with the involved extremity.

d. If it is difficult too, descend backwards.

R. Daily activities

a. The therapist will talk to you about daily routines.

S. Home exercise

The principal in to start exercises with high repetitions of relatively low weight. Start with reduced weight bearing exercises such as swimming and stationary bike and progress to elliptical or treadmill.

You should not feel an increase in symptoms or experience a pressure in the knee during exercise. If so, contact your therapist.

You will experience some discomfort (pain or pressure) with the exercises to improve range of motion. You should continue the exercise as tolerated.

Exercise for lateral buttock and intrinsic muscles

In all of the exercises you should feel the contraction in your buttock.

a. Sleep on your belly. Bend your knees to 90 degrees. Push your feet together.

b. Sleep on your belly with knees straight. Now move one entire leg away from the other one.
c. Sleep on your side with knees slightly bent. Now move the upper leg away while the feet are together.
d. Sleep on your side with knees straight. Spin the thigh around an axis longitudinally through the femur.
e. Perform the previous exercise while moving the upper leg up from the ground.
f. Perform previous exercise with elastic band.
g. Perform exercise “d” with elastic band.
h. Sit on a chair. Push your feet together. Feel the contraction in your buttock.
i. Stand. Push your feet together. Feel the contraction in your buttock.
j. Progress to standing on one leg with correct alignment.
k. Progress to resisted activities of the opposite leg while standing on the affected leg.
l. Perform lunges with good alignment of lower extremities.
m. Perform lunges with elastic band.
n. Perform previous exercise while holding a weight.

Exercise for buttock muscles

a. Sleep on your belly with a pillow under pelvis. Bend your knees to 90 degrees. Control the movement of your leg and push the entire leg toward the ceiling.
b. Perform squatting only when you are able to do it without difficulty.
c. progress squatting with holding a weight.

Exercise for Gastricnemius muscle

a. Push the sole of the foot against an elastic band.
b. Stand on your feet and raise your heel.
c. Progress to unilateral heel raise.

Exercise abdominal muscles

a. Pull in your abdominal muscles with functional activities.
b. The therapist may give you some abdominal exercises if needed.

T. Exercise to improve extensibility

Stretching of lateral and anterior parts of thigh

a. Make sure your pelvis is in good position and not tilted forward.
b. While you are on the bed, slide your heel and extend your knee and hip.
c. Sleep on your belly with a pillow under pelvis. Bend your knees at the same time and avoid leg rotation. If it is painful start the movement with thighs apart.
d. Sleep on your belly with a pillow under pelvis. Bend one knee and rotate your thigh so that your leg moves toward the other leg. Avoid leg rotation.

Stretching of back of thigh and foot

d. Sit on a chair. Actively extend your knees while pulling feet toward yourself.

U. Self-mobilization and active/passive exercises

a. Perform self-distraction mobilization technique to decrease pain at rest if the therapist advises.
    i. Sit with a towel under the distal part of thigh in a way that your foot is above the ground. Hold this position for 10 minutes. You can use weights around the foot with the permission from the therapist.
b. Sit on a chair and put your foot on another chair. Put the web spaces of both hands above the patella. Perform oscillatory pushes to the knee in the direction of the ground. You can progress to use a weight instead of the pressure.
c. While using a weight on the knee, try contraction of hamstring without knee movement. After contraction the hamstring is inhibited and stretches more easily.

d. While in the bed, slide your heel to extend your knee and hip. Use a towel to provide gentle overpressure.

e. Extend your knees in sitting position.

f. Use stationary biking

V. Bracing and orthotics

a. If the therapist advices a brace or shoe insert, please use them regularly.

W. Pain control

a. You can use ice to control pain as needed.

b. You can wrap the knee in case of swelling.

X. Neuromuscular training

a. Ask your therapist about sport-specific trainings, drills, running, jumping, and agility exercises. You will receive another note for such exercises.

Perform only the ones that the therapist has marked to avoid confusion.

3-Knee hyperextension syndrome

A. To improve alignment deficit:

a. Unlock/relax knees to reduce hyperextension.

b. Align knees over feet in different movements.

B. Gait

a. Unlock knees if you do and raise your heel when taking off your foot.

b. Do not walk with the sole of foot. Your foot contacts the ground from heel and takes off from toes. So, land softly on the heel.

C. Sit-to-standing

a. Slide forward in chair. Align feet behind knees as hip-with distance. Contract your buttock and front thigh muscles to lift body up and forward out of chair.

b. Do not forget to let your tibia roll over your foot.

c. Avoid hip medial rotation.

d. Avoid pulling knees back to meet body.

D. Ascending stairs

a. Use rail to decrease weight on the affected limb.

b. Contract your buttock and front thigh muscles to lift body up and forward

c. Do not let your thigh rotate medially when you are bearing weight on your feet.

d. Avoid pulling knees back to meet body.

E. Descending stairs

a. Use rail to decrease weight on the affected limb.

b. Do not let your thigh rotate medially when you are bearing weight on your feet.

c. If it is difficult, step down with the involved extremity.

d. If it is difficult too, descend backwards.

F. Daily activities

a. The therapist will talk to you about daily routines.

G. Running

a. First learn to walk with instructions. Then gradually start running in short periods and increase the time of running.

b. Start running on track or chip trail instead of asphalt. Avoid running on concrete on camber.

c. Shift your weight slightly forward while running.

H. Cycling

a. The phase that you extend your hip is more important than the phase that you pull the leg up. So, focus on contracting the muscle.

I. Home exercise

You should not feel an increase in symptoms or experience a pressure in the knee during exercise. If so, contact your therapist. In all of the exercises you should feel the contraction in your buttock.

Exercise for lateral buttock and intrinsic muscles

a. Stand upright with feet apart. Hold your hand on a place to decrease weight on feet. Shift weight on one foot and contract the buttock muscles.

b. Progress to standing on one leg with correct alignment.

c. Progress to resisted activities of the opposite leg while standing on the affected leg

d. Sit on a chair. Push your feet together. Feel the contraction in your buttock.

e. Stand. Push your feet together. Feel the contraction in your buttock.

f. Perform lunges with good alignment of lower extremities.

g. Perform lunges with elastic band.

h. Perform previous exercise while holding a weight.

Exercise for buttock muscles

c. Sleep on your belly with a pillow under pelvis. Bend your knees to 90 degrees. Control the movement of your leg and push the entire leg toward the ceiling.
d. Perform squatting.
i. Perform squatting while holding a weight.

Exercise abdominal muscles

j. Pull in your abdominal muscles with functional activities.
k. The therapist may give you some abdominal exercises if needed.

J. Exercise to improve extensibility

Stretching of back of thigh and foot

e. Sit on a chair. Actively extend your knees while pulling feet toward yourself.
f. Sleep on your back. Flex your hip to 90 degrees. Now extend your knee.
g. Stand in runners' position while your heel is on the ground. Feel the stretch in your leg.

K. Taping

a. Posterior X-taping will be performed based on the therapist's opinion and aims to control hyperextension of the knee. Other forms of taping such as V-form tape may be performed as well. In case of itching, irritation and skin blisters remove the tape or call your therapist.

L. Pain control

a. You can use ice to control pain as needed.
b. Modify activities to reduce stress.

M. Neuromuscular training

b. Ask your therapist about sport-specific trainings, drills, running, jumping, and agility exercises You will receive another note for such exercises.

Perform only the ones that the therapist has marked to avoid confusion.

4-Patellar Lateral Glide syndrome

The therapist may give you 2 more papers related to “Tibiofemoral rotation syndrome” or “Tibiofemoral hyperextension syndrome” and mark the exercises.

A. **To improve alignment deficit:**
   a. Correct your alignment based on cues from the therapist.
B. **Sit-to-standing**
   a. avoid prolonged periods of increased knee flexion (>90 degrees).
   b. When unable to take breaks, use sitting knee extension to decrease time spent in knee flexion.
C. **Ascending stairs**
   a. Use rail to decrease weight on the affected limb.
   b. Contract your buttock and front thigh muscles to lift body up and forward
   c. Do not let your thigh rotate medially when you are bearing weight on your feet.
   d. Avoid pulling knees back to meet body.
D. **Descending stairs**
   a. Use rail to decrease weight on the affected limb.
   b. Do not let your thigh rotate medially when you are bearing weight on your feet.
   c. If it is difficult, step down with the involved extremity.
   d. If it is difficult too, descend backwards.
E. **Daily activities**
   a. The therapist will talk to you about daily routines.
F. **Home exercise**

The therapist may give you 2 more papers related to “Tibiofemoral rotation syndrome” or “Tibiofemoral hyperextension syndrome” and mark the exercises.

Exercise for lateral buttock and intrinsic muscles

a. Perform lunges with good alignment of lower extremities.
b. Perform lunges with elastic band.
c. Perform previous exercise while holding a weight.
e. Perform squatting.
d. Perform squatting while holding a weight.

G. Exercise to improve extensibility

Stretching of lateral and anterior parts of thigh

a. Make sure your pelvis is in good position and not tilted forward.
b. Sleep on your belly with a pillow under pelvis. Bend your knees at the same time and avoid leg rotation. If it is painful start the movement with thighs apart.
c. Sleep on your belly with a pillow under pelvis. Bend one knee and rotate your thigh so that your leg moves toward the other leg. Avoid leg rotation.
d. Sleep near the end of the bed. Flex both knees to your chest. Now release on leg and extend it as far as it goes. Let your thigh move outward but control your leg not to move. You should gradually be able to close and control your thigh actively. Avoid rotation of the leg or slightly point it inward.
e. Sleep near the edge of your bed at your side. Now with your knee straight move the entire leg back and drop it from behind (drop from the bed). You should feel the stretch in lateral side of buttock and thigh.

H. Taping

Posterior X-taping will be performed based on the therapist’s opinion and aims to control rotation of the thigh. Other forms of taping may be performed as well. In case of itching, irritation and skin blisters remove the tape or call your therapist.

I. **Self-mobilization**

Glide your patellar bone to medial side as the therapist teaches you to perform daily.

J. **Pain control**

a. You can use ice to control pain as needed.

b. Modify activities to reduce stress.

K. **Neuromuscular training**

Ask your therapist about sport-specific trainings, drills, running, jumping, and agility exercises. You will receive another note for such exercises.

Perform only the ones that the therapist has marked to avoid confusion.

5-Tibiofemoral accessory hypermobility

The aim of these exercises is to improve your control over your knee. Please contact your therapist in case you cannot remember the training.

A. Proprioception /Balance

a. Weight shift exercise
b. Progressive increase in weight bearing on the involved side
c. Unilateral stance
d. Stand on unstable surfaces (pillows, trampoline, BOSU ball)
e. Catch a ball while standing on the unstable surface
f. Perform activities on slide boards
g. Gradually perform some part of your pre-injury trainings

B. Agility exercises

a. Hopping timed

Start with short bouts of hopping and longer rests. Increase On time and decrease Off time after a while

i. Bilateral lower extremity hopping with support of upper extremity
ii. Bilateral lower extremity hopping without support of upper extremity
iii. Side-to-side bilateral hopping
iv. Back and forth bilateral hopping
v. Box bilateral hopping
vi. V bilateral hopping
vii. Zigzag bilateral hopping
viii. Perform above exercises with unilateral hopping

b. Jumping from short surface

Land on both feet without excessive valgus or varus. Land softly using ankle plantar flexors and allow knee to flex.

i. Jump forward
ii. Jump backward
iii. Jump each side
iv. Progress by increasing the height of the surface

c. Jump up on to surface

Start with shorter surface and increase height when appropriate

d. Ladder drills

C. Running

If you are able to run 2.5 kilometers without an increase in symptoms or swelling, you can begin cutting exercises:

i. Figure of 8 running (begin with large 8 and gradually increase the size of 8)
ii. Zigzag running with soft cuts
iii. Zigzag running with hard cuts
iv. Zigzag running with cut and spin

D. Jumping

Use plantarflexors to assist in landing. Do not land with knee stiff. Land with proper knee alignment in frontal plane.

E. Drills

Start this part only if you can complete cutting drills without pain or swelling.

i. Drills with equipment related to your sport
ii. Partner drills
